# Considerations for biomarker strategies in clinical trials investigating tau-targeting therapeutics for Alzheimer’s disease

**DOI:** 10.1186/s40035-024-00417-w

**Published:** 2024-05-21

**Authors:** Lewis K. Penny, Richard Lofthouse, Mohammad Arastoo, Andy Porter, Soumya Palliyil, Charles R. Harrington, Claude M. Wischik

**Affiliations:** 1 Institute of Medical Sciences, University of Aberdeen, Aberdeen, UK; 2 Scottish Biologics Facility, University of Aberdeen, Aberdeen, UK; 3 GT Diagnostics (UK) Ltd, Aberdeen, UK; 4 TauRx Therapeutics Ltd, Aberdeen, UK

**Keywords:** Biomarker, Tau protein, Clinical trial, Alzheimer’s disease, Cerebrospinal fluid, Blood, Plasma, Neurodegeneration, Positron emission tomography, Magnetic resonance imaging

## Abstract

The use of biomarker-led clinical trial designs has been transformative for investigating amyloid-targeting therapies for Alzheimer’s disease (AD). The designs have ensured the correct selection of patients on these trials, supported target engagement and have been used to support claims of disease modification and clinical efficacy. Ultimately, this has recently led to approval of disease-modifying, amyloid-targeting therapies for AD; something that should be noted for clinical trials investigating tau-targeting therapies for AD. There is a clear overlap of the purpose of biomarker use at each stage of clinical development between amyloid-targeting and tau-targeting clinical trials. However, there are differences within the potential context of use and interpretation for some biomarkers in particular measurements of amyloid and utility of soluble, phosphorylated tau biomarkers. Given the complexities of tau in health and disease, it is paramount that therapies target disease-relevant tau and, in parallel, appropriate assays of target engagement are developed. Tau positron emission tomography, fluid biomarkers reflecting tau pathology and downstream measures of neurodegeneration will be important both for participant recruitment and for monitoring disease-modification in tau-targeting clinical trials. Bespoke design of biomarker strategies and interpretations for different modalities and tau-based targets should also be considered.

## Introduction

After decades of effort and attrition in Alzheimer’s disease (AD) clinical trials [1, 2], we now have the emergence of the first disease-modifying therapies. They have been initially approved through the Food and Drug Administration (FDA) Accelerated Approval Pathway, aducanumab [3, 4] and lecanemab [5, 6]. Both are amyloid beta (Aβ)-targeting monoclonal antibodies, with lecanemab being recently granted traditional FDA approval [7]. Whilst this progress is undoubtedly in part due to the evolution of candidate antibodies tested in clinical trials toward specifically targeting pathological forms of Aβ, there have been many lessons learned along the way, which have contributed to this milestone. Most notably, the utility and incorporation of positron emission tomography (PET) and fluid biomarkers into clinical trial designs has aided these successes by supporting patient selection and target engagement and facilitating monitoring of disease progression. In terms of enrolment, only recently was it revealed that significant numbers of subjects without amyloid pathology were enrolled into amyloid-targeting trials for AD. More than 20% of subjects diagnosed with AD based on clinical criteria, enrolled in amyloid-targeting phase 3 trials (bapineuzumab and solanezumab), were deemed amyloid negative by PET scan [8]. In terms of efficacy, both aducanumab and lecanemab were originally approved based on the effect on a surrogate biomarker endpoint, the lowering of Aβ plaques, which has been established to reduce cognitive declines across anti-Aβ antibody trials [9]. Despite being initially approved on the basis of the surrogate endpoint through the FDA accelerated approval program, lecanemab did meet its primary endpoint in the recent phase 3 trial: it reduced the cognitive decline by 27% as measured by Clinical Dementia Rating-Sum of Boxes, when compared with placebo after 18 months of treatment [6]. It has now been approved under traditional criteria (https://www.fda.gov/news-events/press-announcements/fda-converts-novel-alzheimers-disease-treatment-traditional-approval). The reduction in clinical decline, however, is modest with debate over how clinically meaningful the reduction is [10] whilst adverse effects, including cerebral haemorrhage, oedema and infusion-related reactions, are common [6]. Such side-effects need to be removed, while focus must also turn to other targets that include tau pathology. Tau-targeting therapies are attractive as disease-modifying therapies for AD for the following reasons.

1. *Tau pathology strongly correlates with cognitive decline in AD*. The spatiotemporal pattern of tau aggregation in the human brain is highly characteristic and stereotyped. This progression forms the basis of the Braak staging system for neurofibrillary degeneration in AD. Importantly, this staging strongly correlates with the cognitive decline of AD patients [11, 12].
2. *Tau plays a central role in key pathophysiological features of AD*. Pathologic tau is a direct mediator of pathophysiological features of AD, including but not limited to:
  Microtubular destabilisation and disruption of axonal transport [13]
  Dysregulation of intracellular calcium [14]
  Mitochondrial dysfunction [15]
  Oxidative stress [16]
  Proteasome dysfunction [17, 18]
  Promotion of neuroinflammation [19]
  Degeneration of microglia [20]
  Synaptic dysfunction and loss [21]
  Altered neuronal activity [22]
  Neuronal loss [23].
3. Tau pathology can be disrupted through different therapeutic modalities.

**Fig. 1:**
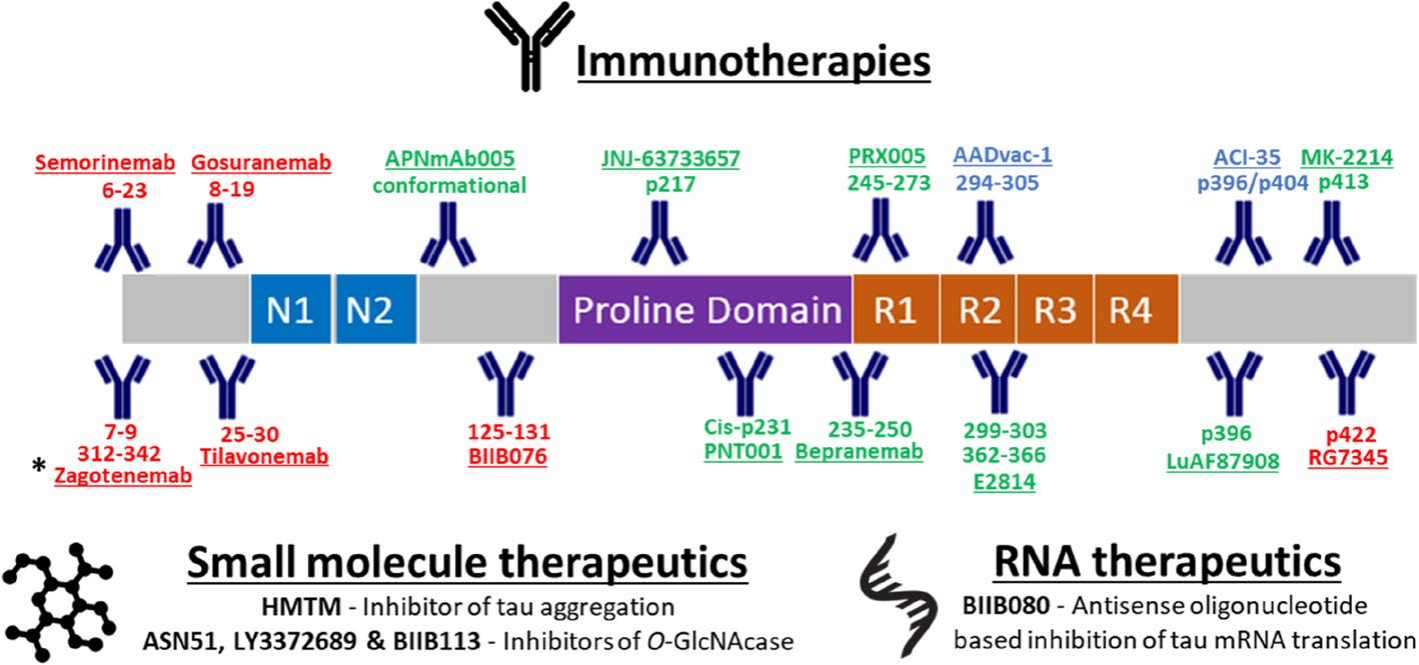
A summary of tau-targeting immunotherapies in clinical development, with their corresponding target regions indicated. Discontinued immunotherapies are shown in red, while passive and active immunotherapies that are in development are shown in green and blue, respectively. * Zagotenemab recognises a conformational epitope involving the N-terminal and repeat region residues. Small-molecule and RNA therapeutics in active clinical development are also shown

Studies in vitro and in vivo have shown evidence that modulation of tau pathology is a viable approach for disease-modifying treatment of AD [24]. The tau targeted therapies in clinical development (Table 1 and Fig. 1) can be mainly categorised into three modalities [25]:

- *Immunotherapies*. Aggregated tau spreads in a prion-like manner from neuron to neuron with a spatiotemporal pattern. As this process is at least in part occurring in the extracellular space [12], tau-targeting immunotherapies are an attractive approach to prevent tau aggregation. Both passive (monoclonal antibodies) and active therapies (vaccines) are in clinical development and they target epitopes across tau including the N-terminus (e.g., semorinemab), the proline region (e.g., bepranemab), the microtubule-binding region (MTBR; e.g., AADVAC1) and specific phosphorylated residues (e.g., ACI-35) [25, 26]. The epitopes recognised by these antibodies are shown in Fig. 1.
- *Protein aggregation inhibitors*. The inhibitors (e.g., hydromethylthionine mesylate) prevent the self-aggregation cascade of tau from the physiologic monomeric form to the pathologic oligomers and neurofibrillary tangles [27, 28].
- *RNA therapeutics*. The RNA therapeutics (e.g., IONIS-MAPTRx, an antisense oligonucleotide, also known as BIIB080) significantly reduce tau expression in the brain to prevent accumulation of tau pathology [29].

**Table 1:** Summary of tau-targeting drugs that are in active clinical development or discontinued (information derived from [25])

Drug name	Synonyms	Therapy type	Tau epitope / target	Trial phase / status	Company
E2814	Not applicable	Immunotherapy (Passive-IgG1)	299–303, 362–366	3	Eisai
HMTM	LMTM, LMTX, TRx0237	Small molecule	Inhibitor of tau aggregation	3	TauRx Therapeutics Ltd
AADvac1	Not applicable	Immunotherapy (active)	294–305	2	Axon Neuroscience SE
ACI-35	VAC20121, JNJ-64042056	Immunotherapy (active)	pSer396, pSer404	2	AC Immune SA-Janssen
JNJ-63733657	Not applicable	Immunotherapy (Passive-IgG1)	pThr217	2	Janssen
Bepranemab	UCB0107	Immunotherapy (Passive-IgG4)	235–250	2	UCB Biopharma
LY3372689	Not applicable	Small molecule	Inhibitor of *O*-GlcNAcase	2	Eli Lilly & Co
Semorinemab	MTAU9937A, RG6100, RO7105705	Immunotherapy (Passive-IgG4)	6–23	2 - discontinued	AC Immune SA – Genentech – F. Hoffman La Roche AG
Gosuranemab	BIIB092,BMS-986168, IPN007	Immunotherapy (Passive-IgG4)	8–19	2 - discontinued	Biogen, (Bristol-Meyers Squibb; iPierian)
Tilavonemab	ABBV-8E12	Immunotherapy (Passive-IgG4)	25–30	2 - discontinued	AbbVie, C2N Diagnostics, LLC
Zagotenemab	LY3303560	Immunotherapy (Passive-IgG4)	Conformational (7–9, 312–342)	2 - discontinued	Eli Lilly & Co
Lu AF87908	hC10.2	Immunotherapy (Passive-IgG1)	pSer396	1	H. Lundbeck A/S
PNT001	Not applicable	Immunotherapy (Passive-IgG4)	*cis*-pThr231	1	Pinteon Therapeutics
APNmAb005	RAA7	Immunotherapy (Passive)	Conformational epitope in early tau oligomers	1	Aprinoia Therapeutics
MK-2214	Not applicable	Immunotherapy (Passive)	pSer413	1	Merck
BIIB080	IONIS-MAPTRx, ISIS 814907	Antisense oligonucleotide	Inhibitor of tau mRNA translation	1	Biogen, IONIS Pharmaceuticals
ASN51	ASN121151	Small molecule	Inhibitor of *O*-GlcNAcase	1	Asceneuron SA
BIIB113	Not applicable	Small molecule	Inhibitor of *O*-GlcNAcase	1	Biogen
BIIB076	NI-105, 6C5 huIgG1/l	Immunotherapy (Passive-IgG1)	125-131	1 - discontinued	Biogen, Neurimmune
RG7345	RO6926496	Immunotherapy (Passive)	pSer422	1 - discontinued	F. Hoffman La Roche AG

Given the importance of biomarker strategies and readouts within past and present amyloid-targeting trials, this review critically considers biomarker strategies to support tau-targeting therapeutics with a focus on lessons learned including similarities with and differences from amyloid-targeting trials.

## Purposes of biomarker strategies are equivalent for tau-targeting and amyloid-targeting clinical trials

Although amyloid-targeting and tau-targeting drugs have different targets, the fundamental reasons for measuring biomarkers across various stages of clinical drug development remain the same.

### Patient screening, selection and stratification

Patient selection is critical for AD trials. An important element is the confirmation of AD diagnosis by amyloid positivity (PET or CSF) with further definition by degree of cognitive impairment, e.g., asymptomatic, MCI due to AD, or mild/moderate/severe AD–related dementia on the basis of criteria set by the National Institute on Aging–Alzheimer’s Association (NIA-AA) [30, 31]. Many recent clinical trials have focused treatment on presymptomatic AD or MCI populations in order to treat the disease as early as possible [32]. This, however, has disadvantages such as higher rates of negative amyloid PET scans in individuals screened for trial entry, larger number of patients to screen and greater costs. It has been reported that approximately 99% of eligible patients are never referred to or consider participating in an AD clinical trial, while the screen failure rates for clinical trials in preclinical AD and MCI due to AD are 88% and 78%, respectively [33]. Sponsors are charged up to $8000 per amyloid PET scan with patient-screening costs accounting for 50%–70% of total per-patient costs in AD trials [33]. The ability of blood-based biomarkers (e.g., Aβ42/40, ptau181, ptau217 and ptau231) to discriminate AD from healthy ageing more than a decade before symptoms develop, allows for cost-effective pre-screening to enrich the population for those who are most likely to have an amyloid-positive PET scan: NCT05026866 (donanemab) [34] and NCT04468659 (lecanemab) [35]. In terms of patient stratification, biomarker strategy can play an integral role in further defining biological stages and identifying subjects who are most likely to benefit from treatment. This may prove pivotal to the success of clinical trials targeting tau protein, but also for understanding the biology and implication of the results. For example, stratifying clinical trial populations by tau pathology (“low” or “high”) will elucidate who will benefit the most from that particular tau-targeting therapeutic (this may be dependent on the specific modality and action of the drug). Again, learnings can be taken from the latest generation of amyloid-targeting therapies, notably donanemab where subjects were stratified by tau pathology as measured by PET, which exhibited a more beneficial clinical outcome in the low/medium tau pathology population vs. the high tau pathology population.

In principle, there may be little to no difference in patient recruitment, selection and stratification strategies between amyloid-targeting and tau-targeting AD trials in terms of biomarker strategy. Clinical trials targeting tau pathology can therefore take advantage of the groundwork already undertaken for trial strategies targeting amyloid pathology.

### Providing evidence of target engagement in early-stage clinical trials

Providing evidence of target engagement as early as possible in clinical development is essential for internal decision making and further development of a therapeutic candidate. This is often part of phase 1b trial designs which offer proof-of-pharmacology and critical information to help determine dosing strategies for subsequent trials. Target engagement for an amyloid-targeting therapy may include a decrease in amyloid PET positivity and/or a decrease in Aβ42/40 ratio in CSF along with an associated increase in blood [36, 37]. Downstream pharmacodynamic biomarkers may also be measured but with the caveat that phase 1 trials are performed with limited numbers of participants over, at least initially, a relatively short duration. Again, in principle, there may be little to no difference in target engagement strategies between amyloid-targeting and tau-targeting AD trials. Tau-targeting clinical trials should incorporate strategies for showing target engagement by measuring disease-relevant tau species in the CSF and blood and by PET scan.

### Pharmacodynamic biomarkers and surrogate endpoints in late-stage clinical trials

In the later stages of clinical drug development (Phase II/III), larger cohorts of patients and longer trials allow for measurement of pharmacodynamic biomarkers (target or downstream) to support clinical endpoints. Although discussed under the primary objective of early-stage trials, confirmation of target engagement and proof-of-pharmacology remains paramount for late-stage drug development. Again, the principles remain the same for amyloid-targeting and tau-targeting therapies. There is a widespread opinion that tau pathology is downstream of amyloid [38], although the clinical evidence for this is mixed [39]. With this in mind, tau-based therapies are unlikely to use measures of amyloid pathology as a potential surrogate endpoint for FDA accelerated approval. However, it is not yet known whether a treatment targeting tau pathology will influence brain amyloid load.

## Are amyloid PET scans necessary for participant recruitment to tau-targeting clinical trials?

The emergence and evolution of amyloid PET over the last decade has been important for the success of amyloid-targeting clinical trials. Firstly, it has ensured that only amyloid-positive AD patients are selected for AD trials and secondly, it can provide surrogate endpoints for efficacy in late-stage clinical trials, a feature that has been instrumental for amyloid-targeting drug approvals by the FDA. The utility of amyloid PET positivity as a requirement for a treatment targeting tau pathology is less clear. Due to the strong and growing body of evidence that blood-based biomarkers such as Aβ42/40, ptau181, ptau217 and ptau231 are powerful in discriminating healthy ageing from AD [40–42], the necessity for expensive amyloid-PET scans in tau-targeting clinical trials is questionable. Of those aforementioned, current best-in-class biomarker for this context of use appears to be ptau217, with continuous evidence of > 95% accuracy in discriminating AD from healthy participants in plasma [43, 44]. In particular, mass spectrometry-based methods of ptau217 maintain this high performance (94.7% accuracy) when identifying the prodromal stages of AD, which are an important population for tau-targeting clinical trials [42]. However, next-generation assays are still coming to fruition and may even outperform ptau217 in terms of discrimination. For example, a novel assay specifically detecting soluble amyloid oligomers in blood can discriminate participants with AD with accuracy as high as 99% when compared to healthy controls [45]. These biomarkers are also selective for AD when compared to other dementias and tauopathies [46–48]. Further studies have shown improved accuracy when information from multiple biomarkers is pooled and modelled with genetic information such as apolipoprotein E (*APOE*) genotype [49, 50]. Although recent evidence has shown that comorbidities such as stroke, chronic kidney disease and cirrhosis may return false positives [51], these conditions commonly exclude subjects from participating in AD trials. Furthermore, covariates such as age, sex and body mass index, can be accounted for within data analyses to improve accuracy [52]. Although amyloid PET positivity has high accuracy in diagnosis, its accuracy is approximately 90% when compared to neuropathological validation [53, 54], an accuracy comparable to that of the best-in-class candidate blood-based biomarkers aforementioned. Furthermore, a review of 15 peer-reviewed studies with a total of 341 patients with AD and 651 subjects with normal cognitive performance, concluded that 96% of the patients with AD had an amyloid-imaging positive result, with a modest specificity of 76% [55]. Given the remarkable progress and use of blood-based biomarkers and tau PET in AD trials (further discussed in following sections) over a short period of time, it seems likely that amyloid PET scans could become non-essential in the near future for tau-targeting therapies.

## Target engagement—lessons learned from targeting the N-terminus

Tau protein is fascinatingly complex in health and disease, and it becomes truncated into multiple fragments, of which the N-terminal fragments are cleaved from the disease-causing core of tau [56]. This has been supported by CSF mass spectrometry data showing fragmentation in disease and abundance of N-terminal fragments in AD [57]. This has led to a number of first-generation tau-targeting immunotherapies targeting the N-terminus such as semorinemab [58], tilavonemab [59], zagotenemab [60] and gosuranemab [61]. However, these antibodies achieved negative outcomes in AD and primary tauopathy clinical trials, showing lack of efficacy and missing their primary endpoints. An exception is semorinemab which, in one of the two phase 2 trials, improved cognitive function (one of the co-primary endpoints) without affecting the activities of daily living or any of the prespecified secondary outcomes [62]. These negative outcomes were achieved despite clear evidence of target engagement, with N-terminal tau decreasing in CSF and increasing in plasma. In the most extreme example, gosuranemab decreased N-terminal tau in CSF by 98% (11% increase for placebo) without showing clinical efficacy in progressive supranuclear palsy (PSP). This finding replicated the results for gosuranemab in tau transgenic mice, in which there was a drastic reduction in CSF N-terminal tau but no difference in mid-region tau [63]. These findings are important for the field and, given the complexity of tau, the first wave of N-terminal antibodies have highlighted that in tau-targeting clinical trials it is integral to not just simply monitor target engagement of ‘tau’ but to measure specifically which populations of tau are engaged. Furthermore, next-generation tau-targeting clinical trials will need to re-focus their target on disease-relevant tau supported with appropriate target engagement assays.

## Ptau181, ptau217 and ptau231 are not exclusive biomarkers for tau tangles

Assays measuring levels of ptau181, ptau217 and ptau231 in both CSF and plasma are capable of discriminating the AD continuum from healthy ageing [42, 64]. However, growing evidence suggests that they may, at least in part, be soluble biomarkers that represent amyloid-induced effects rather than being markers specific for tau pathology. This needs to be considered when evaluating efficacy of tau-targeting treatments. This can be summarised by the following evidence:

1. Initial changes in these biomarkers occur decades before symptoms, correlate with measures of amyloid pathology and precede tau neuropathology [65, 66].
2. Their increase is exclusive to AD. Levels do not change in other tauopathies such as corticobasal degeneration (CBD), PSP, and behavioural variant frontotemporal dementia due to tau accumulation [67].
3. Amyloid-targeting therapies (small-molecule inhibitor ALZ-801 and immunotherapeutic antibodies lecanemab and aducanumab) decrease these biomarkers by removing amyloid [4, 6, 68].
4. In the symptomatic phase of AD, these biomarkers saturate whereas tau pathology advances [69]. This lack of progression over time also makes them less attractive as pharmacodynamic biomarkers regardless of whether they represent, even in part, tau pathology. However, ptau217 does show modest longitudinal progression and correlation with amyloid and tau pathology as determined by PET and neuropathological studies [66].

Although soluble phosphorylated tau biomarkers may not be solely representative of structural tau pathology, they are exclusively upregulated in AD. It therefore remains possible that they have an active role in disease progression and their reduction by tau-targeting therapies could be beneficial for disease modification. However, semorinemab and gosuranemab reduced ptau217 in CSF by up to 27% [62, 70], reductions similar to those seen in amyloid-targeting trials in which clinical deterioration was inhibited. This highlights that this relationship may not be universal across amyloid- and tau-targetted clinical trials. This suggests that further evidence is required to establish what these biomarkers truly represent and their utility as surrogate biomarkers with degree of reduction required in the specific context of each therapeutic modality, epitope and mechanism of action (discussed in further sections of this review). Recent evidence suggests that some phosphorylated tau fluid biomarkers may in part reflect synaptic dysfunction via accumulation and release of oligomeric phosphorylated tau, and also plaque-associated dystrophic neurites that contain oligomeric phosphorylated tau [71, 72].

## Tau PET is critical for biomarker strategies of tau-targeting clinical trials

One of the key advances in this field that will be integral for the evaluation of tau-targeting therapies in AD clinical trials is tau PET. Tau PET offers non-invasive monitoring of the spatiotemporal spread of tau characteristic of the AD continuum. This is highlighted by the FDA approval of flortaucipir tracer for assessing the quantity, localisation and extent of tau neurofibrillary tangles in the brains of those with suspected AD [73]. Previously, this could only have been achieved post-mortem by neuropathological evaluation. In terms of patient recruitment, tau PET will add assurance that those with tau pathology (at any given stage) are included in clinical trials investigating tau-directed therapies. On the contrary, selection of amyloid PET-positive, tau-negative populations may also prove to be a trial population of interest for disease modification in early disease, i.e., preventing the occurrence of tau pathology and progression to AD in high-risk populations. In terms of target engagement and longitudinal disease monitoring, tau PET will likely become as important in tau-targeting clinical trials as amyloid PET in amyloid-targeting trials. The ability to show pharmacodynamic removal or prevention of further spatiotemporal spread of tau pathology will act not only as proof of target engagement but will provide mechanistic support for treatment effects with correlation to clinical improvements. This will be an important element for validation of tau as a druggable target for AD in the clinic. Although tau PET offers advantages of spatiotemporal staging and demonstrates superior correlation with cognitive decline compared to all other biomarkers, its positivity occurs relatively late and closer to symptomatic onset in the AD continuum [74–76]. This allows for the selection of amyloid-positive, tau-negative populations to show disease modification in early disease through tau-targeting therapeutics, i.e. preventing occurrence of tau pathology and progression to AD in high-risk populations. A further consideration is that the tau oligomer load in synapses and subsequent dysfunction play a critical role in AD pathophysiology and clinical decline [77–79]. Given that the currently available tau ligands measure tangle load and not oligomer load, a drug which reduces synaptic tau oligomers may not necessarily reduce tau PET but improve clinical outcomes. It is also of note that tau PET still has great utility in amyloid-targeting trials for participant selection and as a downstream marker of AD pathology [80, 81].

## Emergence of tau tangle-specific fluid biomarkers to support development of tau-targeting therapeutics

There has been encouraging progress in CSF and blood biomarkers that appear to be more specifically representative of tau tangle pathology rather than the amyloid-induced changes, which will be integral for future tau-targeting AD clinical trials. As previously discussed, this is of importance since ptau181, ptau217 and ptau231 appear at least partly related to or induced by amyloid pathology early in the AD continuum and may saturate earlier than markers of structural neuronal damage. Antibody-free mass spectrometric measures of ptau205 in the CSF have identified it as a promising late-stage AD biomarker that progresses in the symptomatic phases of the AD continuum, which may be of interest for tau-targeting AD clinical trials [82]. Another tau biomarker of interest is the decrease of C-terminally truncated tau368 to total tau (t-tau) ratio, which strongly correlates with tau PET and is not influenced by amyloid pathology measured by PET [83, 84]. Importantly, the decrease of CSF tau368/t-tau ratio continues to progress in AD where tau pathology advances and other tau markers reach a plateau [84]. Finally, the decrease of CSF tau368/t-tau ratio outperforms total tau, ptau217 and ptau181 measures in terms of correlation with cognitive performance in symptomatic AD patients [84]. A mass spectrometry-based method for measuring CSF levels of a fragment spanning residues 243–254, termed MTBR-tau243, has been developed. This is of interest to tau-targeting clinical trials given that it increases longitudinally as the AD continuum advances from tau pathology onset, with high correlation to tau PET and cognitive decline, indicating it as a tau tangle-specific biomarker [85, 86]. These assays are limited to the CSF and in terms of blood biomarkers, current measures of ‘total’ tau are of interest but are hampered by the presence of peripheral tau and do not correlate with CSF levels [87]. Recent progress has been made with the development of an assay that specifically measures brain-derived tau that can be measured in blood, correlates with CSF, and is specifically upregulated in AD [88]. This measure is associated with tangle and plaque pathologies at post-mortem, but further research and validation are required to see what it represents, whether it changes longitudinally and whether it may respond pharmacodynamically to tau-directed therapies [88]. Furthermore, recent data suggest that the plasma MTBR-tau243 can be measured with similar characteristics to the CSF MTBR-tau243 [89]. In conclusion, incorporation of both tau PET and biomarkers specific to tau pathology will be useful in current and future tau-targeting clinical trials in terms of participant enrolment and stratification, target engagement and disease-monitoring.

## Bespoke biomarker strategies and interpretations for differing modalities and tau-based targets

It is not within the scope of this article to critically review all tau-targeting therapeutics in drug development. In this section, we highlight that biomarker strategies and the interpretation of their findings may not be universal and will differ between modalities and tau-based targets. The proposed mechanism of action for a tau-targeting drug under investigation needs to be considered when designing a suitably powered clinical trial in which biomarker endpoints are to be used. For example, a tau aggregation inhibitor will slow the longitudinal progression of tau aggregation but may not reduce tau PET signal below baseline because the tangle burden may not be affected. In a similar manner, antisense oligonucleotide approaches that reduce tau expression would be expected to slow the longitudinal progression of tau pathology, as measured by PET, but remain unlikely to drastically affect established tangles. However, in a phase 1b trial investigating the antisense oligonucleotide termed BIIB080 in subjects with mild AD, a significant reduction of tau PET below baseline was observed in a small imaging cohort (*n* = 12) at week 100, likely through reducing the amount of tau protein that can be recruited in the modest ability of turnover in tangles [90, 91]. Furthermore, an immunotherapeutic approach targeting an epitope within tau tangles may aim to acutely reduce tau PET signal below that of baseline under the context of target engagement, but it should be noted that extracellular tangles are a small and limited population to target. It should be stated that all modalities offer potentially valid approaches; and unlike amyloid antibodies that remove plaques as measured by amyloid PET, the removal of tau tangles below baseline as measured by tau PET may not be required for a disease-modifying therapy. Extracellular tau tangles have been referred to as ‘tombstone tangles’, with soluble tau oligomers being the most destructive and toxic tau species, that is, removal of soluble tau oligomers may be more effective than removing tangles [92]. Therefore, interpretation of tau PET results will not be universal and should be done so with the context of use, drug mechanism and bigger picture in mind – an inhibition of longitudinal change of tau PET via removal of soluble oligomers may offer greater effect on clinical outcomes than a significant reduction of tau PET signal and tangles from baseline. In a similar manner for the non-universal interpretation of fluid biomarkers, for example, ptau217 measurements in a trial investigating JNJ-63733657 (a tau immunotherapy targeting ptau217) will no longer be a marker that is a reflective surrogate of tau pathology but of target engagement. Similarly, in the case of an antisense oligonucleotide, the reduction of tau expression will reduce the amount of ptau217 acutely but may not necessarily mean an acute reduction of structural tau pathology. In another example, CSF MTBR assays that are at least in part reflective of tau tangles may no longer be such in the context of certain immunotherapies; the epitope of bepranemab (235–255 AA) [93] is within MTBR-tau243 (measures 243–254 AA fragment [85, 86],) and will likely no longer be exclusively representative of tangles but may suggest pharmacodynamic removal of MTBR-tau fragments and target engagement. In summary, the biomarker analyses in the cases of novel modalities and tau targets will not be universal. Downstream biomarkers of neurodegeneration and changes in clinical syndrome will be critical in validating each approach and interpreting tau biomarker changes for each therapy. This will aid our understanding of the complexity of tau as well as the active and disease-causing component of tau in AD.

## Markers of neurodegeneration are key downstream biomarkers for all tau-targeting therapeutics

In a manner similar to amyloid-targeting therapies showing important downstream decreases in soluble ptau biomarkers in CSF and blood [4, 94], it is important for tau-targeting therapies to show they can affect downstream events and most notably one of the key features of AD, neurodegeneration. This is supported by the NIA-AA Research Framework: “Toward a biological definition of Alzheimer’s disease”, which proposes that neurodegeneration is a downstream consequence of tau aggregation pathology in the AD continuum and when it occurs in the absence of tau pathology, a non-AD-related change is considered [95]. As a minimum, there are two main biomarkers of neurodegeneration that are of interest to tau-targeting clinical trial designs for AD, neurofilament light (NfL, measured in plasma or CSF) and magnetic resonance imaging (MRI).

### NfL

Among the fluid-based biomarkers representing neurodegeneration, NfL is one of the most utilised and promising markers of axonal neurodegeneration [96, 97]. Although NfL levels are elevated in the CSF from AD patients, this is a non-specific marker of neuronal damage. Higher levels of NfL are detectable in other neurological disorders such as amyotrophic lateral sclerosis, acute brain injury (e.g., stroke, traumatic brain injury) and other dementias [98]. In most neurodegenerative diseases, including AD, higher levels of NfL represent faster disease progression and faster rates of brain atrophy [98, 99]. NfL can therefore be regarded as a measure of the intensity of ongoing neurodegeneration. Furthermore, these findings have been validated in blood where NfL levels are elevated in the prodromal [100] and dementia stages [101] of sporadic AD as well as in autosomal dominant AD prior to symptoms [99, 102]. Despite being a promising biomarker candidate for neurodegeneration with utility in tau-targeting clinical trials for AD, it does come with the following considerations for potential use.

#### Patient recruitment

For patient recruitment, NfL is non-specific to AD and is unlikely to have a role in participant recruitment on its own. However, it could be used in combination with amyloid and tau to decipher neurodegeneration-negative or -positive participants on the AD continuum. This could add further clarity on ensuring homogenous participant population or allow for further stratification of subgroups.

#### Monitoring progression of disease and neurodegeneration

For longitudinal disease monitoring, it should be noted that NfL increases even in healthy ageing [103]. Therefore, with even the most effective disease-modifying treatments, an increase from baseline should be expected and comparison to concurrent age-matched controls be required. Furthermore, the rate of increase in plasma NfL concentration in the MCI population is comparable to that observed in healthy controls, but greater in the later stages of the AD continuum. This is important given the more frequent use of the MCI population in tau-targeting trials. For example, in a large cohort study, the plasma NfL concentrations increased with age in the cognitively unimpaired (CU) (2.3 pg/ml per year, *P* < 0.001), the MCI (2.6 pg/ml per year, *P* = 0.43 versus CU), and the AD dementia (5.12 pg/ml per year, *P* = 0.01 versus CU) groups [101]. Even in the dementia phase of AD, these changes in NfL concentration are modest, compared to changes in other neurodegenerative diseases such as PSP and frontotemporal dementia (FTD) [48]. Therefore, relatively large trials over a long period of time (which is often the case for phase II/III clinical trials for AD) are required for NfL to be viable for this context of use in tau-targeting AD clinical trials. Given the relatively modest changes, robust statistical models are required to control for any potential covariates. These could include age, sex, ethnicity, region, *APOE* genotype, disease severity, baseline NfL concentration, baseline clinical disease severity and potentially the use of standard symptomatic AD drugs. Despite these cautions on use of NfL as a longitudinal biomarker of neurodegeneration in AD clinical trials targeting tau pathology, it is encouraging that plasma NfL concentration changes in response to tau-directed therapeutic intervention. Participants with AD receiving AADvac1 treatment (tau-targeting vaccine) in a phase 2 clinical trial (ADAMANT, NCT02484547) had a 12.6% (*n* = 100) increase in NfL from baseline whereas subjects receiving placebo treatment had an increase of 27.7% (*n* = 63, *P* = 0.0046) over the 104-week trial [103]. Although this clinical trial did not meet its primary end points, post-hoc analysis was performed utilising machine learning to predict amyloid- and tau-positive participants from baseline MRI which was used for entry requirement. Participants receiving AADvac1 within this subgroup exhibited a slowing of cognitive and functional decline [104]. It is of interest that the amyloid-targeting antibodies donanemab and lecanemab exhibited disease modification and slowing of cognitive and functional decline without affecting NfL [6, 105]. This may be indicative that amyloid plaque removal can slow the clinical syndrome without directly slowing neurodegeneration. Nevertheless, for evaluating tau-targeting therapies, NfL remains an essential biomarker of downstream neurodegeneration especially given the critical role of tau in neurodegeneration in AD [106].

### MRI

MRI is valuable to AD clinical trials as it captures high-resolution structural images of the brain. These provide information on the whole brain volume, the ventricular volume and regional measures, such as hippocampal volume. It is also a convenient method for use in clinical trials as it is relatively inexpensive and requires no radioactivity exposure and the required scanners are available in most hospitals [107].

#### Patient recruitment

MRI can distinguish AD and MCI patients from cognitively normal controls with relatively high accuracy. However, it is not specific for AD. Evidence of concomitant amyloid pathology is required for AD. For example, substantial hippocampal atrophy is also a feature of hippocampal sclerosis, frontotemporal degeneration, and neurofibrillary tangle-only degeneration [108, 109]. The low sensitivity (73%) and specificity (81%) for predicting MCI patients progressing to AD by MRI, as determined by a meta-analysis, could introduce non-AD patients into clinical trial groups [110]. This is more problematic given the attractive prospect of treating patients as early as possible in the AD continuum in tau-targeting clinical trials and changes measured by MRI being more closely linked to clinical impairment when compared with other biomarkers. Nonetheless, the European Medicines Agency (EMA) has qualified MRI as a biomarker for enriching patients into pre-dementia AD/MCI clinical trials [107]. In summary, MRI may be used to further enrich recruitment of a population with neurodegeneration in clinical trials targeting tau pathology in AD. It should be added that MRI can also support exclusion of patients from tau-targeting clinical trials with confounding pathologies other than probable AD or MCI-AD, e.g., dementia with Lewy bodies, Parkinson’s disease, multiple sclerosis, PSP, hydrocephalus, Huntington’s disease, or prion disorders. Further important exclusions also include significant intracranial focal or vascular pathology seen on brain MRI scans that would lead to a diagnosis other than probable AD or MCI-AD, including but not limited to large confluent white matter hyperintense lesions (i.e., Fazekas score of 3, [111]), other focal brain lesions or evidence of prior or current macrohaemorrhage. The latter pathologies are particularly important for trials targeting amyloid pathology with monoclonal antibodies, given the significant risk of amyloid-related imaging abnormalities associated with this approach [112].

#### Monitoring the progression of neurodegeneration and disease

The rates of whole-brain and hippocampal atrophy are sensitive markers of regionally specific neurodegeneration progression and are being increasingly utilised as outcomes in AD clinical trials assessing disease-modifying therapies [109]. Since MRI provides detailed information regarding brain structure, it can be a more effective method in determining disease-specific neurodegeneration, as opposed to fluid biomarkers that provide global measures of neurodegeneration. MRI has greater consistency when compared to cognitive and functional endpoints, which makes it a valuable tool in AD clinical trials as it can reduce the number of participants required to power for treatment effects [113]. Given the importance of tau-mediated neurodegeneration in AD, MRI has utility in trials targeting tau pathology as exploratory or secondary endpoints and can help provide evidence of disease modification.

It should, however, be noted that changes in regional brain volume have been found to be dynamic in AD [114]. Both cortical thickness and surface area demonstrate positive and inverse correlations for inter-lobar connectivity, which support functional adaptations to and compensations for pathology. These adaptations follow the well-defined functionally linked nodal architecture of the brain and may respond to both a treatment targeting tau pathology and standard symptomatic treatments. Thus, changes in the whole-brain volume produced by AD are not simply a reflection of neuronal death but are subject to functional adaptation and compensation in response to pathology and pharmacological intervention. Furthermore, brain volume measured by MRI is not a simple marker of neurodegeneration and atrophy in the context of amyloid-targeting immunotherapy, where decreases in brain volume are evident despite slowing of clinical decline [80]. This is hypothesized to be due to the rapid removal of protein aggregates and it is unclear whether this will translate to tau-targeting immunotherapies.

#### Safety

MRI can also provide detailed imaging of brain blood vessels, so regulatory authorities require their use in trials targeting amyloid with monoclonal antibodies, as a safety endpoint in AD clinical trials. However, since tau-targeting therapies have not yet been linked with amyloid-related imaging abnormalities, this is unlikely to be a requirement.

## Markers of tau and neurodegeneration as potential surrogate endpoints for clinical efficacy

For amyloid-targeting clinical trials, the FDA deemed that amyloid PET serves as a “surrogate endpoint that is reasonably likely to predict a clinical benefit to patients” [5]. This is supported by the relationship between Aβ plaque lowering and the lower extent of cognitive decline across anti-Aβ antibody trials [9], leading to approval via the FDA accelerated approval pathway. For markers of tau pathology and neurodegeneration in tau-targeting clinical trials, the clinically meaningful level of reduction of change from baseline is unknown and needs to be established. Initial validation will come from different tau-targeting therapies in late-stage clinical development that measure biomarkers of tau pathology and neurodegeneration with correlation to clinical outcomes. These markers could, at least initially, provide supportive evidence for their use as surrogate endpoints in clinical trials targeting tau pathology.

## Don’t forget the X – Novel biomarkers for key processes in AD

It must be remembered that AD is much more complex than its oversimplification to pathological hallmarks of amyloid plaques, tau tangles and neurodegeneration (termed the ATN biomarker system), and AD clinical trials targeting tau pathology will likely need to take account of this. For example, evolving from the ATN framework, Hampel and colleagues introduced the ATXN framework where X represents novel candidate biomarkers of key pathological AD features. These include but are not limited to fluid biomarkers for neuroinflammation (YKL-40, CXCL10), microglial activation (TREM2), synaptic dysfunction (neurogranin, SNAP25 and GAP43), astroglial injury (GFAP, S100B), and blood–brain barrier dysfunction (PDGFRβ) [115]. In addition to these fluid biomarkers, PET tracers and use of MRI for these features of AD are also in development to assess microgliosis, astrocytosis, synaptic density, glucose transport, cerebrovascular function, blood–brain barrier dysfunction, etc. [116–121]. Although these biomarkers will be utilised initially for exploratory purposes, they are important for understanding how patients respond to therapies targeting tau pathology and for enhancing our understanding of the pathophysiological features of AD in relation to the pathophysiology of tau protein in AD.

## Utility of biomarker strategies for therapies targeting tau pathology in clinical trials for primary tauopathies

Although the focus of this review is AD (a secondary tauopathy), many tau drugs targeting tau pathology in AD clinical trials are also investigated against primary tauopathies such as PSP, CBD and FTD. There has been much progress in the field of AD biomarkers including recent advances in translating these to other tauopathies. Similar to AD clinical trials investigating tau-based therapeutics, biomarkers in other primary tauopathies can ensure suitable participant enrolment, provide evidence of target engagement and act as pharmacodynamic biomarkers to support mechanism, efficacy, and disease-modification. This field is in its infancy compared to AD, but clinical trials that incorporate biomarker-led designs need to take account of important differences relative to AD:

- *MAPT* (gene encoding tau) mutations are not linked to familial AD but are associated with a proportion of primary tauopathies. These may help serve as an important biomarker to stratify patients with primary tauopathies to the most appropriate trials.
- Absence of ptau181, ptau217, ptau231 and amyloid biomarkers could help ensure subjects with AD pathology are not enrolled onto these trials.
- Measures of NfL are significantly higher in primary tauopathies such as FTD [122] when compared to AD, which may support participant selection. NfL levels also show greater longitudinal progression [101] in primary tauopathies, which may serve better as a biomarker for tau-targeting drugs in slowing neurodegeneration in these tauopathies.
- With regards to tau PET, the evidence available so far indicates that the currently available tracers bind to the pathological tau filaments of AD. Whether they bind to non-AD tau filament pathology remains inconclusive. This is likely to be due to the molecular differences in pathological tau aggregates [123–125]. Some studies have shown mild tracer uptake in amyloid-negative FTD (^18^F-MK-6240 [126]), PSP (^18^F-T807 [127]), CBD and PSP (^18^F-THK5351 [128]), but further research is required to support these. Given the molecular diversity of pathological tau aggregates in different tauopathies, it is not known whether this represents a limitation of the ligands which are currently available, or whether more suitable ligands can be developed.
- Development of tau biomarkers that are specific to each tauopathy would greatly benefit this field of research. A recent example is the discovery of a peptide comprising tau275-280 in CSF, which is a discriminatory biomarker specific for primary tauopathies, with the highest levels detected in CBD [129]. This biomarker could not only help identify patients for clinical trial enrolment in this rare and difficult-to-diagnose disease, but also respond pharmacodynamically to therapies targeting tau pathology as shown in further research.

In a manner similar to AD, these markers of neurodegeneration and disease-specific tau biomarkers could initially provide supportive and mechanistic evidence for drugs targeting tau pathology if these disease-modifying changes correlate with clinical endpoints.

## Conclusion

The use of biomarker-led clinical trial designs has been transformative for investigating therapies targeting amyloid pathology in AD. They have assisted in patient selection, and supported target engagement as well as claims for disease modification and clinical efficacy. Ultimately, this has led to the FDA approval of disease-modifying amyloid-targeting therapies for AD. This has relevance to treatments targeting tau aggregation pathology. There is a clear overlap of the purpose of biomarker use at each stage of clinical development between clinical trials targeting the amyloid and the tau pathologies of AD, with differences in the role of amyloid PET in the context of use and the use of soluble phosphorylated tau biomarkers. Given the complexities of tau in health and disease, it is important that attention be paid to targeting disease-relevant tau and appropriate target engagement assays need to be developed in parallel. Tau PET and fluid biomarkers of tau pathology or downstream measures of neurodegeneration will be important for participant recruitment in these trials and will prove instrumental in assessing disease-modification in clinical trials targeting tau aggregation pathology.

## Data Availability

Data sharing is not applicable to this article as no datasets were generated or analysed during the current study.

## Abbreviations

AD: Alzheimer’s disease
ATN: Amyloid, tau and neurodegeneration
Aβ: Amyloid-beta
CBD: Corticobasal degeneration
CSF: Cerebrospinal fluid
CU: Cognitively unimpaired
FDA: Food and Drug Administration
FTD: Frontotemporal dementia
MCI: Mild cognitive impairment
MRI: Magnetic Resonance Imaging
MTBR: Microtubule binding region
NfL: Neurofilament light chain
NIA-AA: National Institute on Aging and Alzheimer's Association
PET: Positron emission tomography
PSP: Progressive supranuclear palsy
ptau: Phosphorylated tau
t-tau: Total tau
